# The optical behavior of nano filled resin composite loaded with graphene oxide nanoparticles

**DOI:** 10.1186/s12903-023-03798-y

**Published:** 2024-02-14

**Authors:** Heba fathy, Hassan Haroun, Mona Riad

**Affiliations:** 1 Faculty of Dentistry, Modern University for Technology and Information (MTI), Cairo, 11571 Egypt; 2 Faculty of Dentistry, Cairo University, 11 El-Saraya St, Manial, Cairo, 11553 Egypt

**Keywords:** Graphene oxide nanoparticles, Color change, Spectrophotometer, Nano filled resin composite

## Abstract

**Objectives:**

Assessment of the effect of incorporation of graphene oxide nanoparticles (GONPs), different concentrations into resin composite with different thicknesses on its color modulation.

**Materials and methods:**

GONPs were prepared using the chemical reduction method and characterized using a transmission electron microscope and X-ray diffraction. The minimum concentrations of GONPs that provided the most effective antibacterial action (0.05 wt% and 0.2 wt%.) were prepared to be the concentration added to the tested resin composite. Calculations were done to find the required volume of the GONPs solution needed according to the mass of the resin composite. 70 nano-filled resin composite discs were prepared with 10 mm diameter × 3 mm height. 10 resin composite discs were prepared without GONPs incorporation and served as a control (G0). The other 60 resin composite specimens were divided into 2 equal groups (G1& G2) according to the concentration of the loaded nanoparticles in the specimens. Each group was divided into 3 equal subgroups according to the thickness of the resin composite containing GONPs; [T1: GONPs dispersed in the bottom 1 mm of the disc, while the top 2 mm of the disc was of resin composite only. T2: GONPs dispersed only in the bottom 2 mm of the disc and T3: GONPs dispersed in the total thickness of the disc (3 mm)]. ∆E values were calculated using a Vita Easy shade Spectrophotometer.

**Results:**

Incorporation of GONPs into resin composite induced significant color change and among all the 6 experimental groups, G1T1 group (of 0.05 wt% concentration GONPs dispersed only in the bottom 1 mm of the disc) showed a non-significant color change.

**Conclusion:**

Dispersion of GONPs has a detectable effect on the color change of resin composite. Meanwhile, dispersion in low concentration for only the bottom 1 mm thickness of resin composite has an undetectable effect on its color.

## Background

The primary way of treating caries is filling. Filling needs a variety of materials, including resin composite and adhesives [1, 2]. Dental fillings are subjected to oral environmental fluctuations as saliva, cariogenic bacteria, forces of mastication, humidity, temperature and abrasion, that can result in restoration microleakage brought on by resin shrinkage or adhesive dissolution, fracture, caries recurrence and restoration failure [3, 4]. Therefore, there is always a huge interest and strong trend in continuous development of dental materials with improving properties. The basic approach is to embed antibacterial agents in restorative materials in order to develop anti-biofilm dental adhesives [5, 6]. Supplementation of restorative materials and bonding agents with biomaterials recently tried to prevent restorations failure. Efforts have been undertaken to combine the graphene derivatives with a specialized irrigation technique for endodontic disinfection [7]. Sun et al. 2020 [8] tested Graphene oxide-coated porous titanium as an antibacterial and dentino-inductive restorative material for pulp sealing. The results indicated that the modified titanium-based material by graphene could dramatically up-regulate adhesion, proliferation, odontogenic differentiation, and antibacterial activity.

Graphene-based materials stand out for their exceptional mechanical properties, antibacterial effect, large surface area, high biological compatibility, and advantageous stem cell differentiation [9, 10]. Graphene is a carbon-based plate structure; a single atomic sheet of sp2 (S, Px, Py)-hybridized carbon atoms with a honeycomb lattice arrangement that makes up Graphene the world’s thinnest and strongest substance [11–13]. Graphene has been widely used lately in dentistry because of its distinctive (2D) form, which can be applied in many dental materials, such as resin composite, adhesives, and dental implants [14, 15].

Graphene oxide (GO) is one of the most significant chemical graphene derivatives among various nanomaterials belonging to the graphene family. Graphene oxide (GO) is a compound of carbon and oxygen; the structure of GO is a single-layer plate. GO has been studied as a new biomaterial in dentistry due to its low toxicity and antimicrobial effect against various pathogens [7, 10]. The antibacterial effect of GO, especially on dental pathogens, has been discovered recently, and it has been shown that GO displays the highest antibacterial effect in comparison to other graphene-based materials such as graphite, graphite oxide, graphene oxide, and reduced graphene oxide. Physical damage is induced by blade like graphene materials piercing through the microbial cellular membrane causing leakage of intracellular substance leading to cell death. Wrapping and photothermal ablation mechanism could also provoke bacterial cell damage by enclosing the bacterial cells, providing an unique flexible barrier to isolate bacteria growth medium, inhibiting bacteria proliferation, and decreasing microbial metabolic activity and cell viability [7, 10, 16–18]. So GO have several potential applications in dentistry such as tissue engineering, implants, antibacterial materials, drug delivery carriers, photothermal and photodynamic therapies, and biosensors. Therefore, they have been explored in recent years by numerous researchers worldwide [19–24].

One of the most prominent instances of how nanoscience has significantly advanced dentistry is the development and application of nanomaterials in the field of dentistry [25]. Graphene oxide nanomaterial (GONPs) have high elastic modulus, electronic properties as well as a variety of efficiently fabricating structures, such as graphene quantum dots, nanorobots, and nanotubes. Graphene nanoplates also have an antimicrobial and antibiofilm properties and when combined with polymer materials acts as better dental adhesives with good biocompatibility [26] and have excellent mechanical performance without reducing the adhesive strength [27–29]. Graphene-based nano biomaterials are still in the early stages of development and study, but their special qualities and capacities to act either by themselves or in combination with other biomaterials present a number of potential clinical uses. Since graphene-based compounds effectively suppress the viability of the cariogenic bacteria Streptococcus mutans (S. mutans), they offer reasonable protection against dentin demineralization in restorative dentistry and orthodontics. Graphene Oxide is one of the most important chemical graphene derivatives which possessed a variety of chemically reactive functional groups on its surface, which facilitate connection with various materials including polymers, biomolecules, DNA, and proteins [26]. Therefore, the introduction of graphene-based compounds as nanofillers for dental resin composites and dental adhesives could be a promising strategy to prevent secondary caries and extend service life of the restorations [30, 31].

While the esthetic appearance of resin composite is its main periorty and the ability to match and maintain its color is crucial for aesthetic restorative materials to resemble the appearance of natural teeth that are essential for esthetic success, however, effort in development of GONPs -reinforced composite resin has been hampered because of its dark color, which is a major drawback for esthetic resin composite. The dark discoloration of the resin composite restoration and at dentin–composite resin interface due to the presence of GONPS needs to be overcome to make this application a reality. The aim of this study was to yield all the benefits associated with the use of GO in the resin composite restorative without affecting its esthetic appearance. It seems valuable to evaluate first the effect of loading GONPs on the optical properties of resin composite before other steps of testing the mechanical, physical or biological affect of this addition.

The primary innovation in this work was a trial to mask the dark brown colored sheets of Go nanomaterials that added as fillers to resin composite restorative material through assessing the optical behavior of resin composite loaded with two different concentrations of GONPs to different resin composite thickness. The null hypothesis tested was that there is no difference in the color parameters of resin composite without and that contains 0.05% or 0.2% by wt% concentrations. An alternative hypothesis is that there is no difference in the color parameters between different thicknesses of resin composite (1, 2 and 3 mm) containing GONPs.

## Materials and methods

### Sample size calculation and ethical regulations

The sample size was calculated using power and sample size calculation software (PS, version 3.1.6) [32].The minimally accepted sample size was 9 per subgroup, as the response within each subject group was normally distributed with a standard deviation 0.52. The estimated difference was 0.8 when probability (power) was 0.8. Type I error probability associated with this test was 0.05. The total sample size increased to 10 per sub-group to compensate for a 15% drop out.

### Study design

To study the impact of adding GONPs on the color of resin composite. GONPs were applied to only the bottom (1 mm) leaving the top (2 mm) with resin composite only and to 2 mm leaving the top 1 mm for resin composite only and to the entire thickness of the specimen (3 mm). 10 specimens were prepared from resin composite only and served as a control group (G0). Another 60 specimens were prepared and divided into 2 equal groups (G1& G2) of 30 specimens each, according to the concentration of the nanoparticles. G1: 0.05% wt% and G2: 0.2% wt%. Each group was divided into 3 equal subgroups according to the thickness of the resin composite containing GONPs; [T1: GONPs dispersed in only the bottom 1 mm of the specimen, T2: dispersed in the bottom 2 mm and T3: dispersed in the total thickness of the discs (3 mm)]. Vita Easy shade Spectrophotometer (Vita Zahnfabrik, Bad Sa¨ckingen, Company, Germany) was used to detect the ∆E value after resin composite curing.

### Preparation and characterization of GONPs

All the chemicals used in this investigation were purchased from Sigma-Aldrich Inc. (St. Louis, Missouri, USA), and were an analytical quality (purity: 95%). The synthesis and characterization of engineered GONPs occurred in the Institute for Nanoscience and Nanotechnology, Kafr El sheikh University, Egypt. GONPs were prepared in ethanol solution using the chemical reduction method by treatment of graphite powder with 360 ml of KMnO_4_ and 40 ml of concentrated H_2_SO_4_/H_3_PO_4_ solutions.

After stirring the mixture for 12 h at a temperature of up to 50 °C, it was cooled to room temperature. The mixture was filtered using polyester fiber (Carpenter Co., U.S.A). After centrifuging the filtrate, the supernatant was decanted away. To create the powder of GONPs, the remaining solid material was successively washed with 200 mL of water, 200 mL of 30% HCl, and 200 mL of ethanol for each wash. The supernatant was then decanted away and dried [33]. GONPs powder were dispersed in ethanol to obtain solution with 0.03 gm./ml concentration stored in dark and sealed container [34].

GONPs were characterized using a transmission electron microscope (TEM) (JEOL JEM-2100, Tokyo, Japan.) and X-ray diffraction (XRD) (XPERT-PRO., U.S.A). With the aid of specialized software, data were gathered, and an absorption rate curve was drawn. The TEM was used to record size, shape and particles’ distribution [35]. An XRD pattern has been performed using a powder diffractometer system, with 2Ɵ between (20° − 80°) at a wavelength (Kα) = 1.54614° to identify the structure of the cellular units (d-spacing) used for the confirmation of a successful GONPs synthesis.

### Specimens’ preparation and calculation of the GONPs volume and resin composite mass

**Fig. 1:**
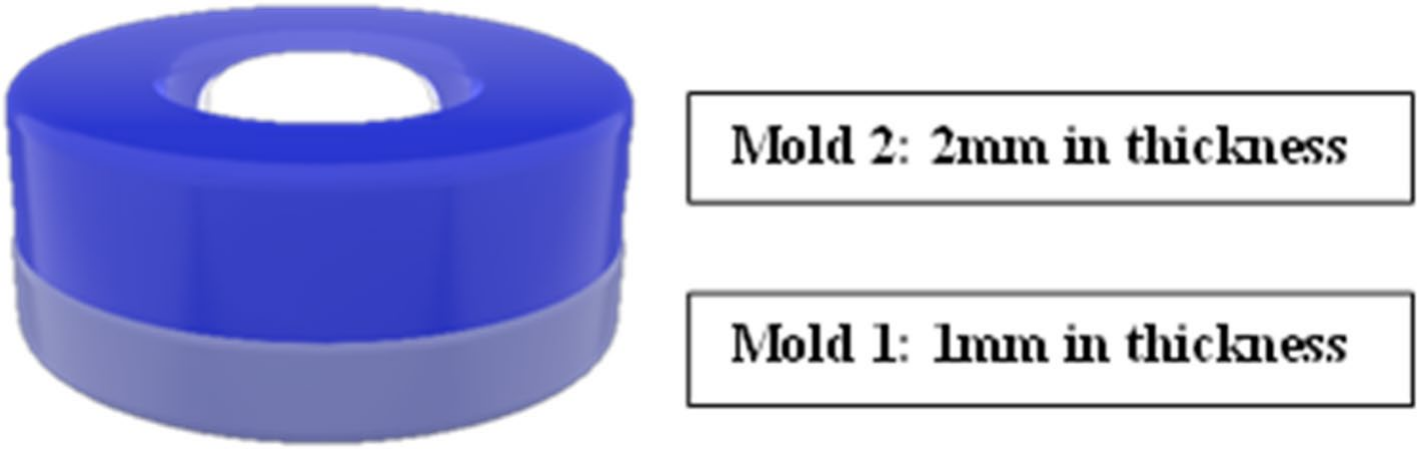
3D model illustration for the molds used for specimen preparation

Two Teflon molds were used to prepare a standardized 70 specimens. Both molds are 10 mm in diameter and differ only in thickness where mold 1 was 1 mm in thickness and mold 2 was 2 mm **(**Fig. 1**)**. Mold 1 was placed on a glass slide and the resin composite restorative material (Table 1) was packed into the mold hole. A mylar strip and another glass slide were applied and pressed over the surface. The excess was removed before curing using a light emitting diode (LED) Curing Unit (3 M ESPE: Elipar deep cure-s., U.S.A) of wavelength range (*λ*) = 430–490 nm, and maximum light intensity *I* ≈ 500 m W/cm^2^, for 20 s according to manufacturer instructions. The cured resin composite was removed from the mold and weighted using a sensitive digital balance. The minimum concentrations of GONPs that provided the most effective antibacterial action of (0.2 and 0.05%) were prepared to be the concentration added to the tested resin composite. Calculations were done to find the required volume of the GONPs solution needed according to the mass of the resin composite of the specimen.

**Table 1:** Materials’ specification, composition, manufacturer and batch number

Material	Specifications	Composition	Manufacturer	Batch number
**Filtek Z250XT Universal Restorative (A2 Body)**	Visible light Cured Nano-filled Resin Composite	Resin: (Bis-GMA)^a^, (UDMA)^b^, (BIS-EMA)^c^, and (TEGDMA)^d^. Fillers: (82 wt %) Zirconia/silica, non-agglomerated/ non-aggregated 20- nanometer surface-modified silica particles.	3 M ESPE St. Paul, MN 55,144 − 1000 USA https://www.3 m.com	NC02944

^a^BIS-GMA: Bisphenol A diglycidylmethacrylate

^b^UDMA: Urethane dimethacrylate

^c^BIS-EMA: Bisphenol A polyethylene glycol diether dimethacrylate

^d^TEGDMA: Triethyleneglycoldimethacrylate

### Weight calculation of GONPs

The cured resin composite was removed from the 1 mm thickness mold and weighted using a sensitive digital balance and it was found to be 0.15 g.

$$\mathrm{Concentration}\;\mathrm{per}\;\mathrm{weight}\;\times\;\mathrm{actual}\;\mathrm{weight}\;\mathrm{of}\;\mathrm{the}\;\mathrm{disc}$$

$$\mathrm{First}\;\mathrm{concentration}:\;0.05\%\times\;0.15\;=\;0.000075\;\mathrm g$$

$$\mathrm{Second}\;\mathrm{concentration}:\;0.2\%\times\;0.15\;=\;0.0003\;\mathrm g$$

The weight of GO needed for a 1 mm disc of composite resin (**T1**) was 0.000075 g for the first concentration (0.05%wt) and 0.0003 g for the second concentration (0.2%wt), subsequently for (**T2**) was 0.00015 g, 0.0006 g and for (**T3**) was 0.000225 and 0.0009 g.

Calculating the volume of GONPs in ethanol was as follows:

$$\mathrm{Volume}=\mathrm{Mass}\;/\;\mathrm{concentration}\;\mathrm{of}\;\mathrm{the}\;\mathrm{solution}$$

$$\mathrm{Volume}\;\mathrm{of}\;\mathrm{first}\;\mathrm{concentration}:\;0.000075/0.03=0.0025\mathrm{ml}\cong2.5\;\upmu\mathrm L$$

$$\mathrm{Volume}\;\mathrm{of}\;\mathrm{second}\;\mathrm{concentration}:\;0.0003/0.03=0.01\;\mathrm{ml}\cong10\;\upmu\mathrm L$$

The volume of GONPs solution needed to be added for each thickness; [T1: 2.5 µL and 10 µL, T2: 5 µL and 20 µL and T3: 7.5 µL and 30 µL].

**Fig. 2:**
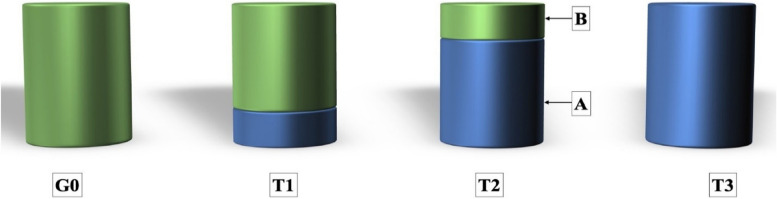
Resin composite disc preparation. **A** resin composite layer containing graphene oxide nanoparticles and **B** Resin composite layer without graphene oxide nanoparticles

The ethanol solution containing GONPs was applied by the micropipette (Piochem. Company, Egypt) into a clean empty amalgam capsule and left until complete evaporation of ethanol which takes about 30 s. The weight of resin composite material previously calculated for the mold 1 was added to the capsule mounted on the amalgamator (YDM-Pro Amalgamator, Hangzhou Yinya New Materials Co. LTD) and mixed for 10 s [36] then packed into the mold and curd in the same way as previously mentioned. Mold 2 was applied over 1 and circled together by a 3 cm diameter copper ring; for fixing both molds together to produce a mold of 3 mm thickness. The resin composite was packed into the mold 2 holes and cured (group G1T1). This step was repeated for other groups G1T2, G1T3, G2T1, G2T2, and. G2T3 (Fig. 2). The specimens’ groups were placed in different jars filled with distilled water at room temperature for 24 h. ∆E values were then calculated using a Vita Easyshade Spectrophotometer. The distribution of the nanoparticles into resin composite was evaluated using Environmental Scanning Electron Microscope (ESEM FEI Inspect, USA).

### Color assessment

Color assessment was performed using the Vita Easyshade Spectrophotometer Compact [35]. The point of the probe was held at a right angle to the surface, and each disc was measured, and recorded [37]. The Commission Internationale de l’Eclairage Lab (CIE L*a*b) color system has been used to determine color difference. The L* coordinate is a measure of the lightness-darkness of the specimen. The specimen’s lightness-darkness is gauged by the L* coordinate. The lighter the specimen, the greater the L* [38]. The red-green axis is measured by a* coordinate. A specimen’s degree of redness or greenness is indicated by a positive or negative a* where a positive a* relates to the amount of redness, and a negative a* relates to the greenness of a specimen. The b* coordinate is a measurement along the yellow-blue axis; a positive b* corresponds to the specimen’s degree of yellowness, while a negative b* corresponds to its degree of blueness. The differences in the CIE color-space parameters are L, a, and b.

∆L, ∆a and ∆b are the differences in the CIE color-space parameters where, $\varDelta L=: L2 - L1; \varDelta a= a2 - a1; \varDelta b= b2 - b1$ [34]. The color of the materials was determined by the difference (ΔE) between the coordinates obtained from the samples at baseline and after graphene incorporation by different concentrations and in different resin composite thicknesses. ΔE was calculated by the following equation: $\varDelta \text{E}=\sqrt{{\left(\text{L}1-\text{L}2\right)}^{2}+{\left(\text{a}1-\text{a}2\right)}^{2}+{\left(\text{b}1-\text{b}2\right)}^{2}}$.Clinically acceptable color difference values that cannot be visually perceivable are when ΔE*≤ 3.3 [38].

### Statistical analysis

Statistical analysis was performed with SPSS 20®, Graph Pad Prism®and Microsoft Excel 2016 ®. All data were explored for normality by using Shapiro-Wilk Normality test and presented as means and standard deviation (SD) values. Comparison between more than 3 different groups (quantitative data) was performed using the One-Way ANOVA test followed by Tukey`s Post Hoc test for multiple comparisons. A comparison between 2 different groups was performed using an independent t-test.

## Results

**Fig. 3:**
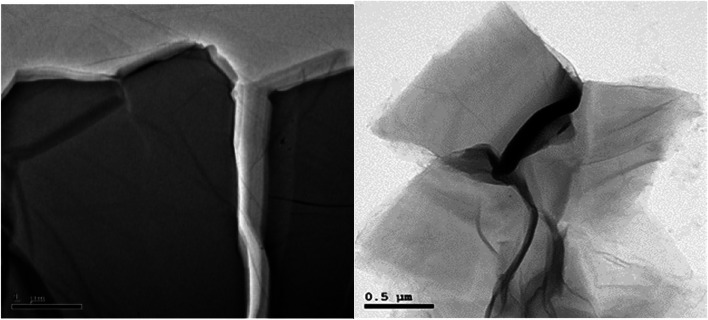
Shape of graphene oxide nanoparticles under TEM

**Fig. 4:**
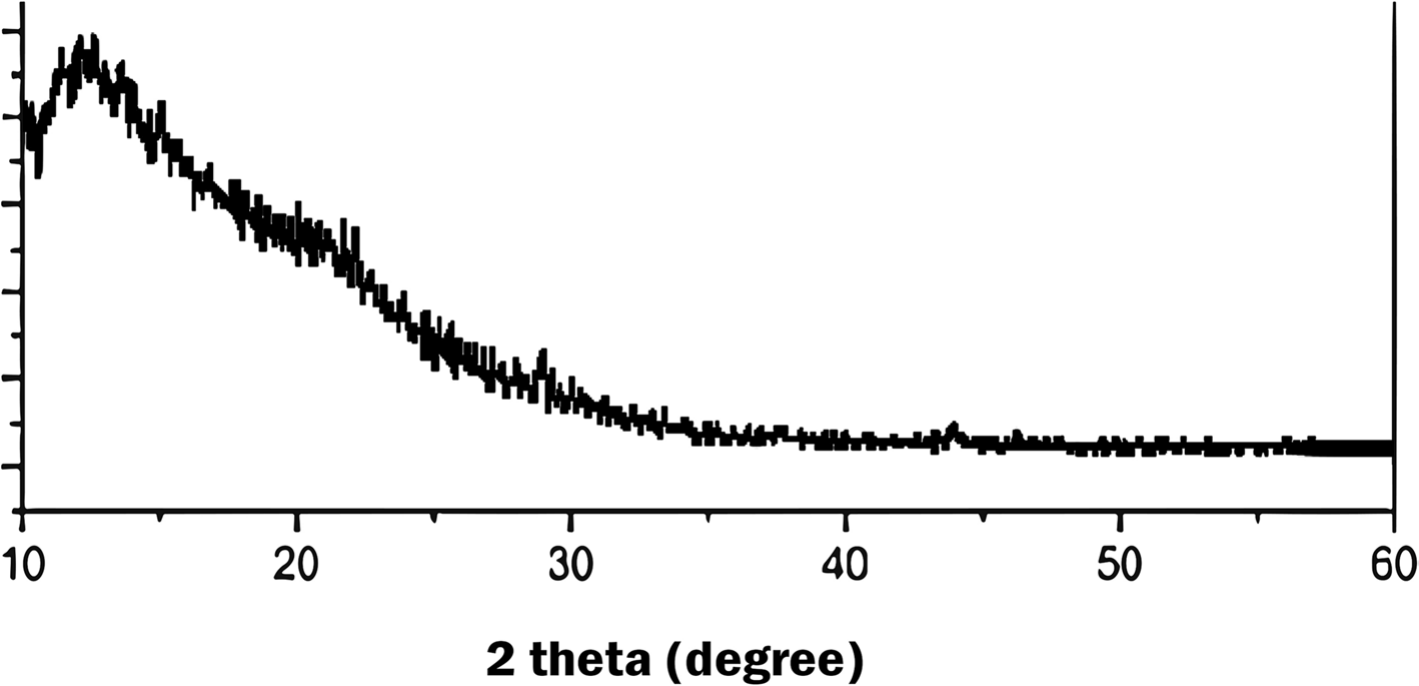
XRD pattern of the absorption rate curve of GONPS

**Fig. 5:**
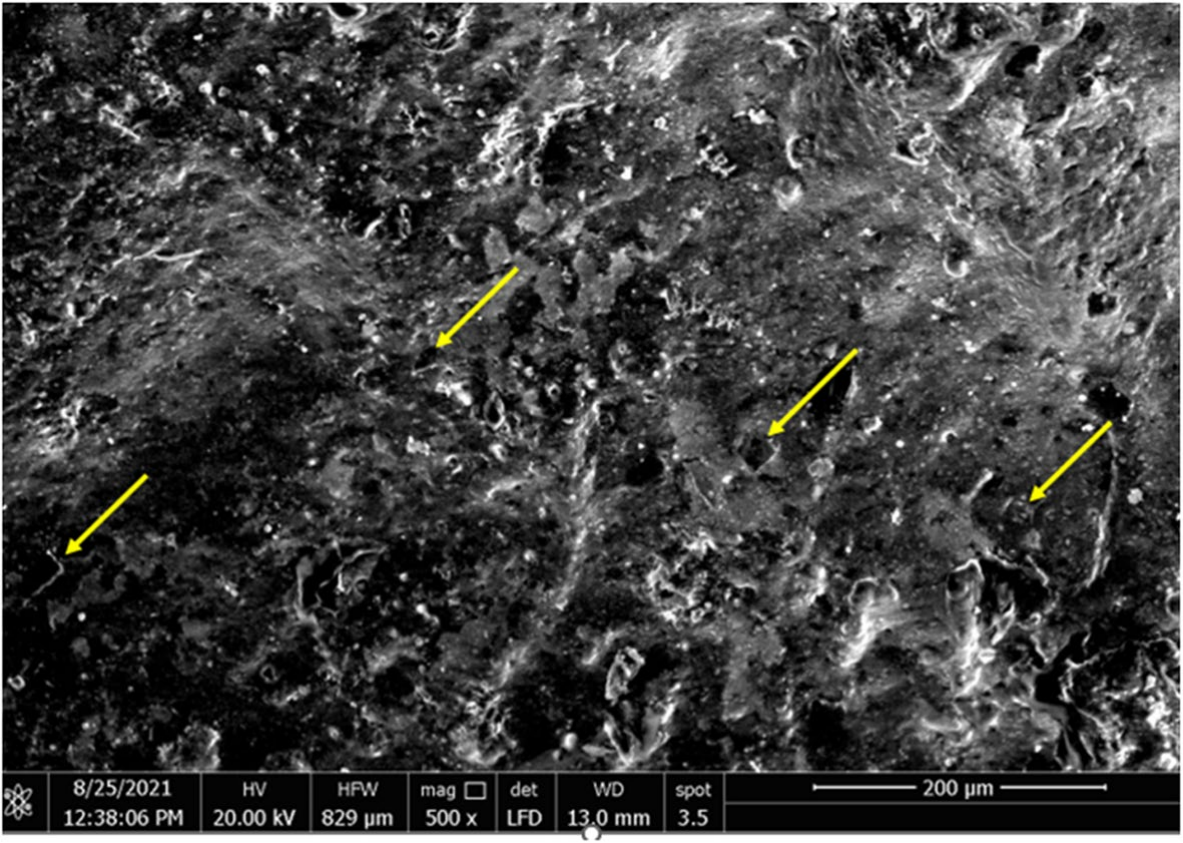
ESEM image of resin composite surface containing Graphene oxide nanoparticles

Visual properties of prepared GONPs revealed that the resultant color of GO solution was dark brown. The TEM analysis of synthesized GO particles demonstrated 1–2 nm thick flakes (Fig. 3). By X-ray diffraction Analysis (XRD), the main reflex of the nanoparticles appeared at 10.3° equal to 0.858 nm confirming the successful GONPs sheets synthesis with an inter- planer distance between the sheets (Fig. 4). Environmental Scanning Electron Microscope (ESEM) In ESEM micrographs (Fig. 5), in terms of dispersion, uniform distribution of GO nanoparticles throughout the resin composite material was observed. The image showed well diffusion of GONPs without agglomeration.

### Color assessment results

The mean values ± standard deviations (M ± SD) of color change values and all CIE color space coordinate parameters (ΔE, ΔL, Δa and Δb) were summarized in Table 2. G1T1 recorded the lowest color change value (ΔE = 1.41 ± 0.99) whereas G2T3 recorded the highest significant color change value (ΔE = 22.01 ± 2.90). There were noticeable color changes in all groups (ΔE ≥ 3.3) except for G1T1 group, which recorded non-significant differences in color values that cannot be visually perceivable (ΔE*≤ 3.3).

The CIE color coordinate differences of resin composite (ΔL, ΔA and ΔB) after graphene incorporation were recorded in minus indicating decrease in luminosity (ΔL < 0) and an increase in green and blue factors respectively (ΔA and ΔB < 0). G1T1 recorded significantly the lowest ΔL, Δa, and Δb values and significantly the highest values were recorded for G2 T3. Concerning ΔL values, there was significant difference among all groups except for G1T2, G1T3, G2T1 and G2T2, and they recorded non-significant differences between them. Concerning Δa and Δb values, there were significant differences among all groups except for G1T2 and G1T3 they recorded non-significant difference between them.

**Table 2:** Mean and standard deviation of ΔL, Δa, Δb and ΔE among all groups (one-way ANOVA)

**Group**	**ΔE**		**ΔL**		**Δa**		**Δb**		***P*** **value**
	M	SD	M	SD	M	SD	M	SD	
**G1 T1**	1.41^a^	0.99	-0.13^a^	0.014	-0.03^a^	0.028	-1.4^a^	0.098	0.0001*
**G1 T2**	3.39^b^	1.07	-1.1^b^	0.4	-0.22^b^	0.027	-3.2^b^	0.099	
**G1 T3**	3.67^b^	0.60	-1.76^b^	0.31	-0.29^b^	0.07	-3.21^b^	0.072	
**G2 T1**	6.08^c^	0.60	- 0.86^b^	0.35	-1.17^c^	0.17	-5.9^c^	0.01	
**G2 T2**	8.78^d^	0.48	-1.46^b^	0.65	-1.65^d^	0.11	- 8.5^d^	0.29	
**G2 T3**	22.01^e^	2.90	-19.84^c^	1.42	-3.15^e^	0.25	-9 ^e^	1.28	

*M *mean*, SD *standard deviation*, p=0.05. NS *non-significant, *=Significant

Legends (a, b, c, d, e) represent the ranking between different tested groups where (a) denoting the lowest values and (e) the highest

### Effect of graphene incorporation on color parameters differences

Table 3 shows a comparison of total (ΔE, ΔL, Δa and Δb) results (M ± SD) as a function of GONPs incorporation in 2 different concentrations in resin composite restorative material. Regardless of the resin composite thickness, it was found that specimens incorporated with GONPs with higher concentration (G2) (0.2 wt%), recorded statistically significant higher color change values (ΔE). Groups containing 0.05 wt% GONPs recorded non-significant differences in color values that cannot be visually perceivable (ΔE*≤ 3.3).

The CIE color coordinate differences of resin composite (ΔL, Δa and Δb) decreased significantly with increasing GONPs concentration (G2) indicating a decrease in luminosity and an increase in green and blue factors respectively.

**Table 3:** Comparison between different groups in ΔE, ΔL, Δa and Δb as a function of GONPs powder incorporation in 2 different concentrations in resin composite restorative material (independent t-test)

M ± SD	G1 With Graphene Oxide Nanoparticles (0.05 wt%)	G2 With Graphene Oxide Nanoparticles (0.2 wt%)	*P*-value
ΔE	2.82 ± 0.89	12.29 ± 1.33	0.0001*
ΔL	-1.12 ± 0.27	-5.96 ± 0.49	0.0001*
Δa	-0.66 ± 0.07	-1.51 ± 0.23	0.0001*
Δb	-2.6 ± 0.09	-7.8 ± 0.53	0.0001*

### Effect of resin composite thickness containing GONPs on color parameters differences

Table 4 shows a comparison of total (ΔE, ΔL, Δa and Δb) results (M ± SD) as a function of resin composite thickness containing GONPs (T1, T2 and T3). Regardless of the GONPs concentration, increasing resin composite thickness incorporated with GONPs significantly increases the color change value (ΔE) (T1 < T2 < T3). The CIE color coordinate differences of resin composite (ΔL, Δa and Δb) decreased significantly with increasing resin composite thickness incorporated with GONPs indicating a decrease in luminosity and an increase in green and blue factors respectively (T1 < T2 < T3).

**Table 4:** Comparison between different groups in ΔE, ΔL, Δa and Δb as a function of the thickness of resin composite layer incorporated with GONPs (T) (one-Way ANOVA)

M ± SD	T1 (1 mm)	T2 (2 mm)	T3 (3 mm)	*P*-value
ΔE	3.74 ± 0.79^a^	6.09 ± 0.78^b^	12.84 ± 1.75^c^	0.0001*
ΔL	-0.62 ± 0.27^a^	-1.31 ± 0.33^b^	-9.19 ± 0.39^c^	0.0001*
Δa	-0.12 ± 0.015^a^	-0.73 ± 0.12^b^	-2.4 ± 0.18^c^	0.0001*
Δb	-3.65 ± 0.054^a^	-5.85 ± 0.1945^b^	-6.105 ± 0.676^b^	0.0001*

* =Significant

## Discussion

The introduction of nanometer-sized particles is one of the most recent developments in the field [25, 39]; which are thought to provide superior wear resistance and strength in addition to enhanced aesthetics and perishability [40, 41]. The desire to take advantage of the ability of nanoparticles potential to alter the structure of the composite has sparked an increase in interest in nanotechnology and its application in resin composites. This might enhance the resin composite’s mechanical, chemical, and optical qualities, enabling it to function perfectly throughout the mouth [27–29, 41, 42]. As a result, the unique nano filled resin composites were subsequently marketed as the Filtek range of restorative materials (3 M ESPE, St Paul, MN, USA) [43]. Several clinical trials for review intervals of one to ten years revealed that this nanocomposite has good surface features, and color match, with no detection of restoration failure and postoperative sensitivity [43–45]. Reduced filler plucking and wear and improved polishability and surface gloss retention [46–48].

As graphene-based dental biomaterials have good systemic and local biocompatibility with human cells [10], so can be safely incorporated with resin composite. GONPs were selected in the current study as they exhibit the strongest antibacterial impact [49]. It has been reported that the 2D nano-flakes (Fig. 2) can encircle the bacterial cell to prevent nutrient uptake. Additionally, they possess jagged edges and act like nano-knives, piercing and rupturing the cell membrane [8, 18, 26, 30, 31]. The concentrations of 0.05w.% and 0.2w.% were previously proven to have effective antibacterial action against the most cariogenic bacteria; *Streptococcus mutans* [29]. GONPs significantly increase the resin composite’s fracture toughness and polymer’s surface hardness and generate better mechanical reinforcement [49, 50]. Incorporation of GONPs can also limit the molecular mobility by the interaction between nano-fillers and polymer matrix and improve the coefficient of thermal expansion and contraction [26, 29]. It has a uniform distribution in the resinous matrix as confirmed by ESEM analysis (Fig. 5) and can also disperse uniformly without agglomeration inside the polymer matrix [29].

Compared to visual color determination, instrumental color analysis may have an advantage since instrumental readings are objective, quantifiable, and more rapidly obtainable. Various techniques have been employed to objectively ascertain color changes on composite restorations. One such technique is spectrophotometry, which enables the examination of multiple factors associated with the color stability of composite resins. In this method reflected wavelength by a body is changed in values expressed in ΔE* units. The ΔE* values can be used so that present the color changes provided by the composite resin after treatment or period of time.

The synthesized nano-GO particles were flake shaped, as observed via TEM in the study (Fig. 3). The reduction process of graphite to yield GO nanoparticles produces flake shaped particles or sheets with sharp edges due to the presence of oxygen groups in the oxide sheets, as reported previously [29]. It is essential that the foreign filler particles be evenly distributed in the resin composite in order to utilize maximum benefit of the addition. The optimal performance of the resin composite may be diminished by gaps or cracks resulting from an incomplete or partial diffusion of filler nanoparticles [11]. The homogeneous dispersion of nano-GO particles was revealed by SEM examination in the current investigation (Fig. 5).

In this work, the impact of adding GONPs to resin composite specimens of varying thicknesses (1, 2 and 3 mm) in two distinct concentrations (0.05 and 0.2 wt%) on its color modulation was examined. Regardless of the resin composite thickness, it was found that specimens incorporated with GONPs with high concentration (group G2) (0.2 wt%), recorded statistically significant higher color change values (ΔE), while the low concentration caused a change in the ΔE* but the change was non-significant (less than 3.3) and not perceivable visually. The null hypothesis was that there is no difference in the color parameters of resin composite without and that contains 0.05w.% or 0.2w. % concentrations. The null hypothesis was Partially accepted as the incorporation of GONPs with concentration of (0.05 w.%) in resin composite caused a non-signigicant colour change from the control group.

The mass fraction of GO influences the light transmittance characteristics of the composites [39], where the resin composite with 0.05 wt% exhibits the highest transparency. However, increasing the concentration of GO from 0.05 to 0.2wt% gradually reduces the light transmittance of materials. The gradual loss of light transmittance could be resulted from the GO color, which limits light penetration during photo-polymerization.

The reflected color of the resin composite significantly affected by the existence of a background. The base material under the restoration, discolored tooth tissue, integration of additives like metals and metal oxides, or the surrounding environment can all be considered a background of different colors of white, black, brown, or other light trapping materials [51]. As graphene is a 2D allotrope of crystalline carbon with unique optical properties have the objectional color of dark brown [10, 19–21] as confirmed in the current study and can be considered a dark colored background. With the presence of dark background, the color measurements (CIE ΔL, Δa and Δb *) were shifted (From red to green; negative ∆a*, from yellow to blue: negative ∆b*). All the specimens showed loss of luminosity (negative ∆L*). The color, thickness and surface properties of a background should be identified when the color of a solid specimen is measured [51, 52]. CIE a* and b* parameters were changed due to the influence of background reflection as it produces chromatic changes of the resin composite final restoration ( 32, 51, 52). These findings were confirmed by other researches which proved that incorporated different metal and metal oxide NPs like silver NPs into dental biomaterials in a way to improve their mechanical and antimicrobial properties and demonstrated color changes of resin composite restorations that might be related to color of nanoparticles [53–56].

The light refraction and reflection at the interface between the matrix and filler are affected by the differences between the inorganic nanoparticles and organic matrix refractive indices. The greater the refractive index difference, the greater the resin composite opacity irrespective of NPs type [56]. Additionally, others proved that the addition of metal oxide nanoparticles like titanium oxides and zinc oxides might decrease color changes and the optical properties of light-cured resin composite [57]. Zhihao Li et al. 2023 [39] synthesized ZnO nanorod-decorated graphene oxide (GOn@ZnO) particles and their optical property was regulated by changing the amount of seeded GO (n value) in the microemulsion. Among all hybrid particles, GO3@ZnO exhibited a bright gray color and lowest UV absorbance and therefore was selected as an optimal functional filler to produce dental composites with different loadings (0.1, 0.5, 1, and 3 wt %). The introduction of GO3@ZnO in dental composites could be a promising strategy. Data on the effect of loading GO NPs on the optical performance of resin composite restoration are lacking, making comparison of our study results difficult.

Additives incorporation to resin composite can change the refractive index of the matrix and increase the perceived light scattering making the material less translucent [51, 52, 58]. These additives besides increasing the darkness of the background, they might agglomerate and change the macroscopic surface features as the surface roughness that greatly affect the optical properties of resin composite. Additionally, the findings of increased darkness of resin composite when loaded with high concentration of GONPs also agree with Lee and others [51] who revealed that if the filler size is constant, the higher filler load might produce a rough resin composite surface with subsequently reflected light scattering in different angles that lowers the translucency with a more darkening effect [58, 59].

Regardless of the GONPs concentration, our study verified that there were significant differences in the color change of resin composite as color change value (ΔE) increased with increased resin composite thickness containing GONPs. The alternative hypothesis was that there is no difference in the color parameters between different thicknesses of resin composite (1, 2 and 3 mm) containing GONPs.

The alternative hypothesis was partially accepted as there was a non significant difference in the color parameters between specimens containg GONPs in the base 1 mm thickness and control group. Increasing the resin composite thickness containing GONPs causes color change. A 1 mm Graphene oxide Nano-sheets resin composite thickness covered by 2 mm of resin composite allow visible light transmission, making no obstacle to the light passage so not reflecting its dark color through the resin composite to the observer eyes [60]. The thin unique 2 D sheets structure can disperse uniformly without agglomeration inside the polymer matrix (Fig. 5) making uniform color distribution inside the resin composite.

With increasing GONPs concentration (0.2 wt%) and decreasing the thickness of the resin composite top layer that non-containing graphene (T2, T3), the ΔE results increased significantly, which may be strongly affected by two important optical phenomena; translucency of the resin composite with and without GONPs and the masking ability. The ability of a substance to transmit visible light is known as translucency [61]. In the current study, decreasing the thickness of the bottom layer of GONPs containing resin composite diminishes the effect of the dark background and enhances the translucency of the whole specimen. This might be in accordance with the researches that reported that the color of a solid specimen is inversely affected by increasing thickness of the background ( 51, 52). It was believed that the presence of dark background necessities opaquer to mask its deleterious effect on color change [32, 60, 62, 63]. Opaquer is highly pigmented resinous material containing metal oxides like aluminum or titanium oxide that have opacification ability as one of the techniques to decrease the effect of dark background beyond the resin composite [32, 60, 62, 63]. The disadvantage of opaquer is handling errors in controlling its thickness which may cause a decrease in the full restoration translucency and increase the deviation of resin composite color significantly [64].

Masking ability of the resin composite restoration is the tendency to mask the background discoloration [32, 51]. In the current study, increasing resin composite thickness mono-shade over composite containing 0.05% GONPs up to 2 mm significantly lower the ΔE. This finding was in agreement with Haas et al. 2017 [52] where in order to reduce translucency and conceal or remove the dark background effect, one of the most crucial variables is to increase resin composite thickness. The finding follows the Beer–Lamberts law where the light transmitted is a function of thickness and the transmission coefficient of the material [52, 57], and for optimal esthetic resin composite results, the background should be of a minimal thickness (0.6-1 mm) and covered by minimally 2 mm resin composite thickness to mask the background effect [62]. As a general, whenever the ∆ E calculation is ≤ 3.3, the color discrepancies can be considered clinically acceptable as it will not be visible to the human eye, despite the values of CIE* instrumental color differences (∆a, ∆ b, and ∆L). Calculating ∆E is the clinical judging factor whether the color of the final restoration is affected by the incorporation of GO NPs or not despite the noticeable changes in other color difference parameters (∆a, b, and L*).

This study is unique because it is the first to assess the addition of graphene oxide nanoparticles to resin composite. As the esthetic appearance of resin composite is prime and first concern so decision to study first if the incorporation of GONPs will not cause color modulation for the resin composite, and in case of acceptable results, the effect on other properties as physical, mechanical and biological will proceed. This study fills a gap in the existing research by exploring the potential impact of graphene oxide nanoparticles as a filler to other materials. The findings from this study will contribute to a better understanding of the potential applications of graphene oxide nanoparticles in dental materials.

Although the developments and researches of graphene based biomaterials related to dentistry are still at infancy, their adorable properties and their abilities to functionalize alone or combined with other biomaterials offer several opportunities in possible clinical applications. Some very promising properties of the biocompatible GO have to be extensively investigated both in vitro and in vivo. GONs is a rather fascinating material worthy of in-depth investigation. We hope that this article could provide some valuable elicitation for the future scientific and technological innovations of graphene in dentistry.

The current investigation is limited by the fact that it was a pure laboratory study.Considering that oral cavity is a complex environment, inability to simulate the clinical scenario to evaluate the optical characteristics of resin composite immediate and after aging. Although instrumental color analysis was employed, visual determination of color change could add more information.

## Conclusions

The incorporation of GONPs in low concentration (0.05 wt%) in resin composite caused a limited and undetectable effect on its optical properties with a clinically acceptable value (ΔE*ab ≥ 3.3). Meanwhile, color change arises with increasing both the concentration (0.2w.%) and the thicknesses of the layer of resin composite containing Graphene Oxide NPs where resin composite showed color change above the clinically accepted value. On a clinical and academic level, color harmonization of resin composite can be preserved by enforcing graphene oxide nanoparticles in low concentration and only in the first packed layer of the resin composite in the created cavities. The experimental material analyzed in this study extend modern research trends in the development of dental materials.

## Data Availability

The datasets used and/or analyzed during the current study are available from the corresponding author on reasonable request.
